# Evaluation of Actinium-225 Labeled Minigastrin Analogue [^225^Ac]Ac-DOTA-PP-F11N for Targeted Alpha Particle Therapy

**DOI:** 10.3390/pharmaceutics12111088

**Published:** 2020-11-12

**Authors:** Yun Qin, Stefan Imobersteg, Alain Blanc, Stephan Frank, Roger Schibli, Martin P. Béhé, Michal Grzmil

**Affiliations:** 1 Center for Radiopharmaceutical Sciences ETH-PSI-USZ, Paul Scherrer Institute, 5232 Villigen, Switzerland; yun.qin@psi.ch (Y.Q.); stefan.imobersteg@psi.ch (S.I.); alain.blanc@psi.ch (A.B.); roger.schibli@pharma.ethz.ch (R.S.); 2 Department of Chemistry and Applied Biosciences, ETH Zurich, 8093 Zurich, Switzerland; 3 Division of Neuropathology, Institute of Pathology, University of Basel, 4031 Basel, Switzerland; stephan.frank@usb.ch

**Keywords:** actinium-225, cholecystokinin B receptor (CCKBR), minigastrin analogue PP-F11N, targeted alpha particle therapy (TAT)

## Abstract

The overexpression of cholecystokinin B receptor (CCKBR) in human cancers led to the development of radiolabeled minigastrin analogues for targeted radionuclide therapy, which aims to deliver cytotoxic radiation specifically to cancer cells. Alpha emitters (e.g., actinium-225) possess high potency in cancer cell-killing and hold promise for the treatment of malignant tumors. In these preclinical studies, we developed and evaluated CCKBR-targeted alpha particle therapy. The cellular uptake and cytotoxic effect of actinium-225 labeled and HPLC-purified minigastrin analogue [^225^Ac]Ac-PP-F11N were characterized in the human squamous cancer A431 cells transfected with CCKBR. Nude mice bearing A431/CCKBR tumors were used for biodistribution and therapy studies followed by histological analysis and SPECT/CT imaging. In vitro, [^225^Ac]Ac-PP-F11N showed CCKBR-specific and efficient internalization rate and potent cytotoxicity. The biodistribution studies of [^225^Ac]Ac-PP-F11N revealed CCKBR-specific uptake in tumors, whereas the therapeutic studies demonstrated dose-dependent inhibition of tumor growth and extended mean survival time, without apparent toxicity. The histological analysis of kidney and stomach indicated no severe adverse effects after [^225^Ac]Ac-PP-F11N administration. The post-therapy SPECT-CT images with [^111^In]In-PP-F11N confirmed no CCKBR-positive tumor left in the mice with complete remission. In conclusion, our study demonstrates therapeutic efficacy of [^225^Ac]Ac-PP-F11N without acute radiotoxicity in CCKBR-positive cancer model.

## 1. Introduction

Targeted radionuclide therapy (TRT) delivers cytotoxic radioactivity specifically to cancer cells through tumor-targeting molecules. To date, β^-^ particle-emitting radionuclides have been routinely employed for the development of TRT against malignant tumors. Lutathera (lutetium-177 oxodotreotide) for neuroendocrine tumors [1], Zevalin (yttrium–90 Ibritumomab Tiuxetan) for non-Hodgkin’s follicular lymphoma [2], and AZEDRA (iodine-131 iobenguane) for advanced or metastatic pheochromocytoma or paraganglioma [3] represent FDA-approved β-particle-emitting pharmaceuticals. TRT is a promising therapeutic modality for patients with disseminated diseases, non-operative tumors, as well as tumor metastasis. As compared to standard-of-care chemotherapy and external beam radiotherapy, TRT significantly reduces toxic effects and improves efficacy through selectively targeting the receptors or antigens, which are exclusively present or overexpressed on the cancer cells. Although β-particle-emitting pharmaceuticals have shown benefits in the treatments of heterogeneous tumors, they may also cause damage to the surrounding healthy cells due to the long tissue penetrating range (e.g., up to 2.1 and 12 mm for lutetium-177 and yttrium-90, respectively) [4]. Furthermore, the moderate linear energy transfer (LET) of β-particles (0.4–2.2 keV/μm) can also result in insufficient DNA damage, which leads to the activation of DNA-repair mechanisms and radio-resistance [4]. Alpha particle emitters with a more confined scope of radiation range and high LET have been explored as prospective radionuclides for TRT. Xofigo (radium-223 dichloride) is the first FDA approved α particle-emitting radioactive therapeutic agent for the treatment of castration-resistant prostate cancer (CRPC) patients with bone metastases and has demonstrated extension in overall survival (OS) and delay in time to first symptomatic skeletal event (SSE) as compared to placebo [5]. Actinium-225 is another promising α particle emitter with a half-life of ten days. It possesses high LET of 80 keV/μm and causes large amount of DNA double-strand breaks (DSB), leading to high cytotoxicity in targeted cancer cells, whereas unfavorable radiation of actinum-225 to the surrounding healthy tissue is diminished due to its short penetration range in tissue (40–100 μm) [6]. Kratochwil et al. reported a proof-of-concept clinical study with [^225^Ac]Ac-PSMA-617, actinium-225 in conjugation with a small molecular ligand of the prostate-specific membrane antigen (PSMA), which was highly effective in the treatment of CRPC patients after failure of [^177^Lu]Lu-PSMA-617 therapy [7].

The overexpression of cholecystokinin B receptor (CCKBR) has been previously found in different types of tumors, including medullary thyroid carcinoma (MTC), stromal ovarian cancer, small-cell lung cancer, and astrocytoma [8,9,10]. The expression of CCKBR was also identified in gastroenteropancreatic tumors, leiomyomas and leiomyosarcomas [11] indicating wide application prospects of the radiolabeled minigastrin analogues for diagnosis and therapy of the CCKBR-positive cancers. Currently, clinical studies including a phase 1 and 0/1 clinical trial of [^111^In]In-CP04 and [^177^Lu]Lu-PP-F11N for diagnosis and therapy of advanced MTC (NCT03246659 and NCT02088645, respectively) are ongoing. The most recent clinical data showed favorable biodistribution and pharmacokinetic properties of β-particle-emitting [^177^Lu]Lu-PP-F11N and indicates stomach as a dose-limiting organ [12]. Minigastrin analogue PP-F11N acts as an agonist ligand of the CCKBR. After binding to the CCKBR, receptor-bound radiolabeled PP-F11N undergoes internalization and the cytotoxic radiation is delivered into the cancer cells [13]. This makes PP-F11N potentially an attractive radiopharmaceutical for delivery of alpha particle emitters.

The aim of the present study was to assess the therapeutic potential of targeted alpha particle therapy (TAT) by using [^225^Ac]Ac-PP-F11N in the CCKBR-positive preclinical tumor model. Furthermore, we compared the biodistribution of [^177^Lu]Lu-PP-F11N and [^225^Ac]Ac-PP-F11N. Our data demonstrate desirable biodistribution and therapeutic efficacy in A431/CCKBR tumor-bearing nude mice without toxic effects and thus, provides preclinical evidence for the development of safe and efficacious CCKBR-targeted α particle therapy (TAT).

## 2. Materials and Methods

### 2.1. Radiolabeling, Purification and Quality Control of Radiolabeled Peptides

N-terminal DOTA-conjugated gastrin analogue PP-F11N (DOTA-(DGlu)_6_-Ala-Tyr-Gly-Trp-Nle-Asp-Phe) and Leu-(Glu)_5_-Ala-Tyr-Gly-Trp-Met-Asp-Phe peptides were purchased from PSL GmbH (Heidelberg, Germany), whereas [^225^Ac]Ac (in 0.1 M HCl) and [^177^Lu]LuCl_3_ solution from ITG GmbH (Munich, Germany) and ^111^In[In]Cl_3_ was supplied by Curium Netherlands BV (Petten, NL). For Ac-225 labeling, 6 MBq [^225^Ac]Ac (12 pmol) and 60 nmol of PP-F11N were mixed in 120 µL of 0.4 M ammonium acetate buffer (pH 5.5), 21 µL of 0.5 M sodium ascorbate was added and the total volume was 510 µL. The labeling was carried out at 75 °C for 1 h. For Lu-177 labeling, 1:30 [^177^Lu]Lu/PP-F11N ratio (20 MBq [^177^Lu]Lu (27.7 pmol) per 830 pmol PP-F11N) was prepared in 0.4 M ammonium acetate buffer (pH 5.5) containing ascorbic acid in a final volume of 50 µL. The labeling was carried out at 90 °C for 15 min. For SPECT imaging, 23.4 nmol of PP-F11N and 120 MBq [^111^In]In (69.5 pmol) were prepared in 133 µL 0.4 M ammonium acetate buffer (pH 5.5), 66 µL 0.5 M ascorbic acid was added and the labeling was performed at 95 °C for 20 min. After labeling, 2 µL of 0.5 mM EDTA in metal free water was added to complex any free metals. Radiolabeled PP-F11N was separated from the unlabeled PP-F11N and free radionuclides using a Merck Hitachi LaChrom 2D high-performance liquid chromatography (HPLC) system, equipped with a D-7000 interface, a L-7200 auto sampler, a radiation monitor (RM-19, EBERLINE Instrument Corporation, SANTA FE, NM, USA), a UV detector (Pharmacia LKB-UV-M II), a 515 Waters pump, a L-7100 Hitachi pump and a Dr. Maisch stability 120 BS-C23 cartridge (5 µm, 10 × 4.6 mm) connected with a reversed-phase column (Dr. Maisch stability 120 BS-C23, 5 µm, 250 × 4.6 mm). The cartridge and the column were purchased from Morvay Analytik GmbH, Basel, Switzerland. Elution was done by the use of H_2_O:0.1% TFA (A) and Acetonitrile:0.1% TFA (B) linear gradients with 32–90% B over 30 min at a flow rate of 1 mL/min. The eluted fractions, which contained purified radiolabeled peptide, were evaporated on SpeedVac and diluted in PBS for in vitro and in vivo experiments. The specific activity of [^225^Ac]Ac-PP-F11N was 475 MBq/nmol and the average chemical purity was analyzed by thin layer chromatography (TLC) and was above 92% (Supplementary Figure S1a). The average radiochemical purity of Lu-177 and In-111-labeled PP-F11N was analyzed by HPLC and was above 99% (Supplementary Figure S1b), and the specific activity was 714 and 1726 MBq/nmol, respectively.

### 2.2. Cell Culture

Human squamous carcinoma A431 stable cell line that overexpresses CCKBR (A431/CCKBR) was generated and kindly provided by Dr. Luigi Aloj [14]. Cells were cultured in Dulbecco’s Modified Eagle Medium (DMEM), supplemented with 10% (*v/v*) fetal bovine serum (Bio Concept Ltd., Allschwil, Switzerland), 2 mM glutamine and antibiotics (0.1 mg/mL streptomycin, 100 IU/mL penicillin and 1.25 μg/mL fungizone) at 37 °C in a humidified incubator containing 5% CO_2_.

### 2.3. Internalization Assay

On the 6-well plates, 1 × 10^6^ A431/CCKBR cells per well were cultivated overnight. On the next day, PBS-washed cells were incubated with 160 Bq of purified [^225^Ac]Ac-PP-F11N (0.33 pM) or 12.3 kBq of purified [^177^Lu]Lu-PP-F11N (171 pM) in 1 mL DMEM with 0.1% BSA at standard tissue culture condition for two hours. 4 μM LEEEEEAYGWMDF peptide was used for blocking experiments. After incubation, supernatant (together with 2x PBS wash solutions) was collected. Then, the cells were incubated in 0.05 M ice-cold glycine buffer (pH = 2) for 5 min for twice followed by a lysis step in 1 M NaOH for 15 min at 37 °C. All three collected fractions (supernatant/PBS; glycine solution; lysed cells) were measured on a Packard Cobra II Auto-Gamma counter (PerkinElmer, Schwerzenbach, Switzerland); energy range for Lu-177 and Ac-225 (daughter nuclides) was 15–600 and 75–500 keV, respectively. Internalized and membrane-bound fractions are shown as % of the total activity. Unspecific membrane binding (glycine fraction) or internalization (NaOH-dissolved cells) from the experiments with blocking peptides were subtracted from the obtained results.

### 2.4. Proliferation Assay

On the 96-well plates 4 × 10^3^ cells/well were seeded. Next day, different radioactivity levels of [^225^Ac]Ac-PP-F11N (0.01 to 316.23 kBq/mL, 2.07 x 10^−5^ to 0.654 pmol) were added to the A431/CCKBR cells. After 2 h incubation, medium containing unbound [^225^Ac]Ac-PP-F11N was removed and the cells were incubated for another 24 h in fresh medium. Cell proliferation was analyzed using CellTiter 96 AQueous Non-Radioactive Cell Proliferation Kit (Promega AG, Dübendorf, Switzerland) according to the manufacturer’s instruction. Absorbance of formazan product was measured at 570 nm with a reference of 650 nm using a MicroPlate Reader (PerkinElmer, Schwerzenbach, Switzerland). The absorbance of the control (untreated) cells was set as 100% cell viability. The activity level of [^225^Ac]Ac-PP-F11N resulting in 50% cell viability was calculated and presented as the half-maximal effective activity (EA_50_) level in cell-killing. The assay was performed in triplicate.

### 2.5. Animal Studies

All experiments were performed in accordance with Swiss Animal Protection Laws. In particular, the animal studies were approved by the Cantonal Committee of Animal Experimentation (License No. 75700, October 2017). Athymic immunocompromised CD-1 female nude mice were purchased from Charles Rivers, Sulzfeld, Germany.

For the biodistribution studies, 2 × 10^6^ of A431/CCKBR cells in 0.1 mL of sterile phosphate-buffered saline (PBS) were injected subcutaneously (s.c.) on the left and right flank (two tumors per animal) of the anesthetized nude mice. 12 to 14 days after implantation, the nude mice carrying A431/CCKBR tumors of approximately 0.1–0.2 cm^3^ were intravenously injected with 38 kBq HPLC-purified [^225^Ac]Ac-PP-F11N (0.08 pmol) or 150 kBq HPLC-purified [^177^Lu]Lu-PP-F11N (0.21 pmol). 1, 4, 24, 48 h and 7 days after the administration of radiolabeled peptides, the mice were sacrificed and the post mortem dissected tumors and organs were weighted and their activity was measured on a gamma counter (Packard Cobra II Auto Gamma, PerkinElmer, Schwerzenbach, Switzerland).

In the therapy studies, the nude mice were s.c. injected with 5 × 10^6^ A431/CCKBR cells on the left shoulder. 5 to 7 days after tumor implantation, the nude mice carrying A431/CCKBR tumors of approximately 0.1–0.2 cm^3^ were randomly grouped and intravenously injected with 30, 45, 60, 90 and 120 kBq (0.06, 0.09, 0.12, 0.18 and 0.24 pmol) of HPLC-purified [^225^Ac]Ac-PP-F11N in 100 µL PBS. The control group was injected with 100 µL PBS. The tumor diameters and mice weight were recorded daily during the working days. The tumor volume was calculated by using the formula V = (W^2^ × L)/2 [15]. The nude mice were sacrificed when the tumor volume exceeded 1.5 cm^3^ or when tumor ulceration appeared. An ABS digimatic Caliper (Mitutoyo Corporation, Kawasaki, Japan) was used to measure the tumor diameter. The data were obtained from two sets of experiments including control, 30, 45, 60 kBq and control, 60, 90, and 120 kBq treatment groups.

### 2.6. Histopathology Studies

For histological analysis, the paraffin sections were prepared from the post mortem dissected and formalin-fixed tumors, stomachs and kidneys (3 mice per each group). After deparaffinization and rehydration, the slides were subjected to standard Eosin-Hematoxylin staining according to the user manual and the images were photographed by using slide scanner (Nikon Instruments Europe, Amstelveen, The Netherlands).

### 2.7. SPECT/CT Imaging

Radiolabeled [^111^In]In-PP-F11N was purified by HPLC, concentrated on SpeedVac, diluted to 13 MBq per 100 μL of PBS and intravenously injected into the nude mice (13 MBq/100 μL (7.54 pmol) per mouse). Imaging of [^111^In]In-PP-F11N in A431/CCKBR tumor-bearing nude mice 55 days after treatment with 45 and 60 kBq of [^225^Ac]Ac-PP-F11N was performed by a single-photon emission computed tomography (SPECT) combined with an X-ray computed tomography (multipinhole small-animal Nano SPECT/CT camera, Mediso Medical Imaging Systems, Budapest, Hungary). 2 h after injections, [^225^Ac]Ac-PP-F11N treated mice were imaged with a 7.5 min CT followed by a 45 min SPECT scan (four pyramids with four standard mouse Aperture (Mediso, NSP-108-M14-WB); energy windows: 245–171 keV; number of projections: 180). As a positive control, untreated mouse bearing two A431/CCKBR tumors was subjected to imaging 20 h after [^111^In]In-PP-F11N injection using the same protocol. The image reconstruction and processing was accomplished by using VivoQuant 3.0 Patch1 software.

### 2.8. Statistics

GraphPad Prism 7.00 for Windows (GraphPad Software, San Diego, USA) was used for all statistical analysis. The half-maximal effective activity (EA_50_) of [^225^Ac]Ac-PP-F11N was calculated from the dose–response curve (nonlinear regression). Two-tailed heteroscedastic Student’s *t*-test was performed for two groups in the biodistribution studies, whereas one-way ANOVA combined with Dunnett’s multiple comparison test was used to compare the control and all [^225^Ac]Ac-PP-F11N-treated groups in the therapy studies. Log-rank (Mantel-Cox) test and Gehan-Breslow-Wilcoxon test were performed to compare different survival curves of the treatment groups with the control group. Endpoints were defined as death in survival curves. Values of *p* < 0.05 were considered statistically significant. The results are reported as mean ± standard deviation of at least three independent replicates.

## 3. Results

### 3.1. Internalization and Cytotoxicity of [^225^Ac]Ac-PP-F11N

**Figure 1:**
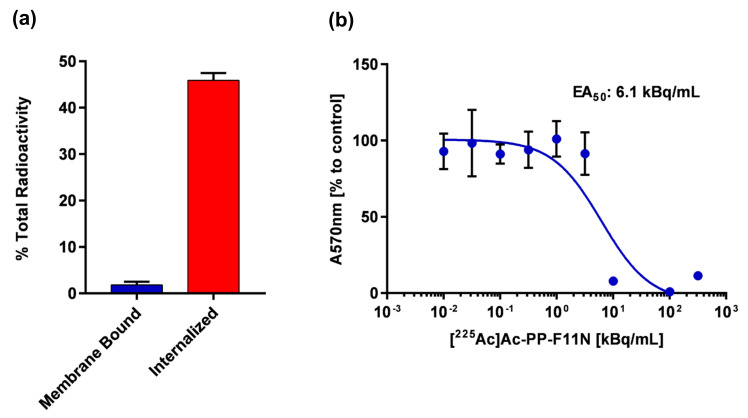
In vitro characterization of [^225^Ac]Ac-PP-F11N in A431/CCKBR (cholecystokinin B receptor) cells. (**a**) Internalized and membrane-bound activity after 2 h treatment with [^225^Ac]Ac-PP-F11N in A431/CCKBR cells. Bars represent mean ± SD. (**b**) Proliferation of [^225^Ac]Ac-PP-F11N-treated A431/CCKBR cells at indicated doses after 2 h internalization followed by 24 h incubation. Points represent mean ± SD. The obtained curve was used for EA_50_ calculation. All experiments were performed at least in triplicate. The SDs at 10^1^, 10^2^, and 10^2.5^ kBq/mL were below 2.6% and are not visible in the figure.

To evaluate the cholecystokinin B receptor (CCKBR)-specific cellular uptake of [^225^Ac]Ac-PP-F11N, the in vitro internalization assay was performed. The internalization rate of [^225^Ac]Ac-PP-F11N reached 45.9% ± 1.6 and the membrane-bound activity was 1.8% ± 0.7 of total activity (Figure 1a). The blocking peptide inhibited internalization rate to 1.4%, indicating CCKBR-specific cellular uptake of [^225^Ac]Ac-PP-F11N. Similarly, internalization rate and the membrane-bound activity of [^177^Lu]Lu-PP-F11N was 44.5% ± 3.0 and 3.1% ± 2.0, respectively (Supplementary Figure S2). To analyze the cytotoxic effect, cell proliferation assay was performed. Half-maximal effective activity (EA_50_) value was calculated and reached 6.2 ± 1.1 kBq/mL at 24 h after [^225^Ac]Ac-PP-F11N treatment (Figure 1b). Maximum cytotoxic effect (0% of cell viability) was reached at 100 kBq/mL, whereas 1 kBq/mL of [^225^Ac]Ac-PP-F11N had no cytotoxic effect on A431/CCKBR cells (100% viability of control cells).

### 3.2. Biodistribution of [^225^Ac]Ac-PP-F11N

**Figure 2:**
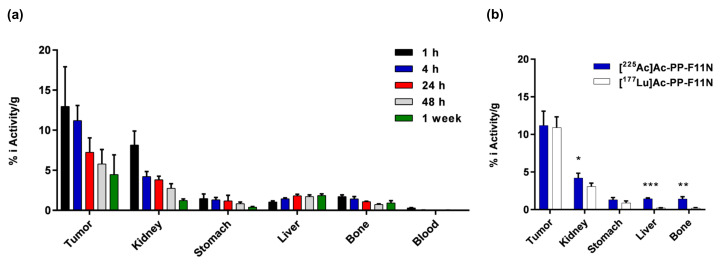
Biodistribution of [^225^Ac]Ac-PP-F11N in A431/CCKBR xenografted nude mice. (**a**) Radioactivity uptake in tumors, kidneys, stomach, liver, bone and blood at 1, 4, 24, 48 h and 7 days after the administration of [^225^Ac]Ac-PP-F11N shown as % of the total injected radioactivity per gram of tissue (% i.A./g). Bars represent mean ± SD, *n* = 4 for each time point. (**b**) Comparative analysis of the radioactivity uptake in indicated organs at 4 h post-injection of [^225^Ac]Ac-PP-F11N (blue bars) and [^177^Lu]Lu-PP-F11N (white bars). Bars represent mean ± SD, *n* = 4. * *p* < 0.05, ** *p* < 0.01, *** *p* < 0.001.

The ex vivo biodistribution studies at 1, 4, 24, 48 h, and 7 days post-radiopharmaceutical application were performed in the A431/CCKBR tumor bearing nude mice. High tumor uptake of [^225^Ac]Ac-PP-F11N was observed at 1 and 4 h post-injection and reached 13 ± 5 and 11.2 ± 1.9% of the injected radioactivity per gram (% i.A./g), respectively (Figure 2a). After 24, 48 h, and 7 days, the tumor uptake decreased as expected to 7.2 ± 1.8, 5.7 ± 1.8 and 4.5 ± 2.5% i.A./g, respectively. Analysis of the healthy organs revealed 1.5 ± 0.6% i.A./g in the stomach at 1 h post-injection (p.i.) due to the endogenous expression of CCKBR [13]. The activity in the stomach remained similar within 24 h (1.3 ± 0.3 and 1.2 ± 0.7% i.A./g at 4 and 24 h) and decreased to 0.8 ± 0.2 and 0.4 ± 0.1% i.A./g at 48 h and 7 days p.i. A 50% decrease of the radioactivity accumulation from 8.1 ± 1.7 to 4.2 ± 0.6% i.A./g was observed in the kidney within the first four hours. The radioactivity level further decreased from 3.8 ± 0.5 to 1.2 ± 0.2% at 24 h and 1 week p.i. In other analyzed organs, the radioactivity reached maximum 0.38 to 0.02% i.A./g at 1 h p.i. and decreased within 1 week to 0.15–0.01% (Supplementary Table S1). Furthermore, we performed comparative biodistribution studies with lutetium-177 labeled minigastrin analogue [^177^Lu]Lu-PP-F11N, which recently has entered pilot and phase I study for the therapy and imaging of metastatic medullary thyroid cancer (NCT02088645). As shown in Figure 2b, [^225^Ac]Ac-PP-F11N showed similar biodistribution profile as [^177^Lu]Lu-PP-F11N at 4 h p.i. The tumor uptake was very similar but moderate higher uptake was observed in the liver and bone as compared to [^177^Lu]Lu-PP-F11N and reached less than 2% i.A./g at all analyzed time points. The additional uptake in liver and bone may be mainly related to free daughter nuclides which are set free by the recoil mechanism. As compared to the tumor-to-kidney ratio and the tumor-to-stomach ratio at 1 h p.i., both ratios increased from 1.59 and 8.85 to 3.64 and 11.49 at 1 week p.i., respectively, which indicated the tumor-specific accumulation of [^225^Ac]Ac-PP-F11N (Supplementary Table S2).

### 3.3. Therapy Study of [^225^Ac]Ac-PP-F11N

**Figure 3:**
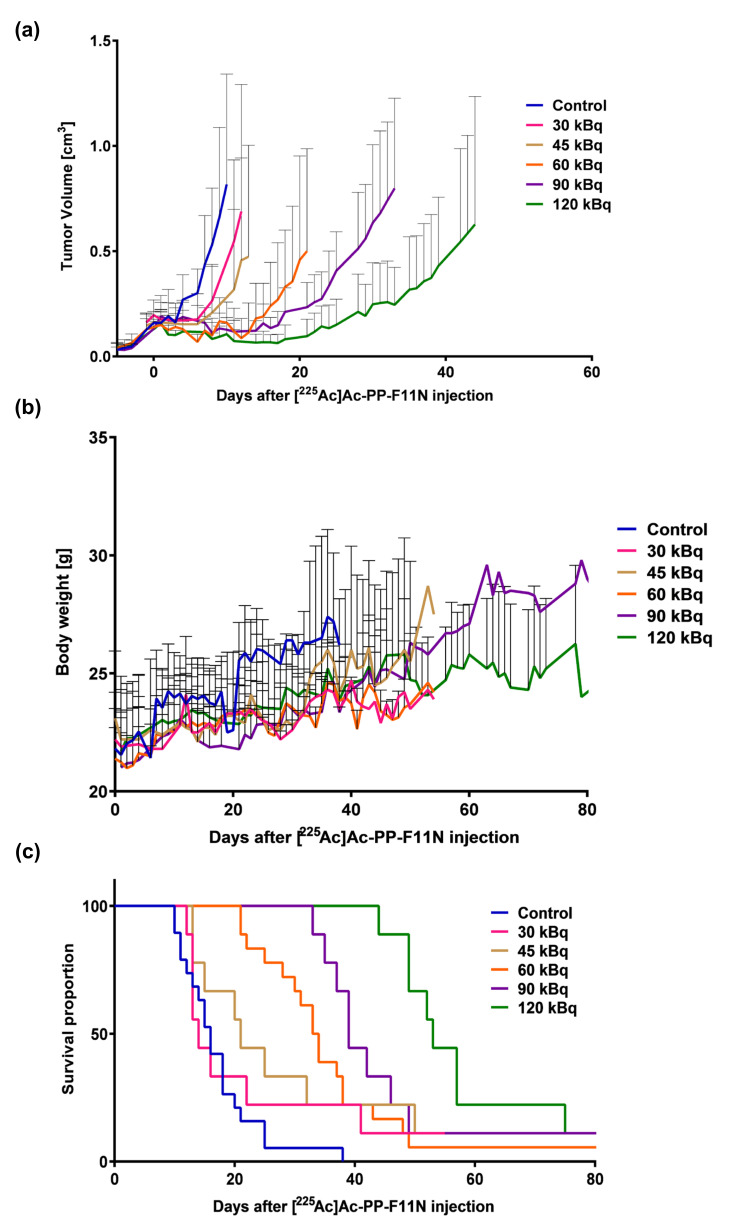
Tumor growth inhibition and prolonged life span in [^225^Ac]Ac-PP-F11N-treated mice. After tumor implantation, PBS or 30, 45, 60, 90, and 120 kBq of purified [^225^Ac]Ac-PP-F11N was administrated into A431/CCKBR tumor-bearing nude mice groups as indicated (*n* = 9, except for the control and 60 kBq group for which *n* = 18). The tumor growth (**a**) and the body weight curves (**b**) in different treatment groups. Values are shown as mean ± SD. (**c**) The survival rates presented as Kaplan-Meier curves of the control and [^225^Ac]Ac-PP-F11N-treated groups.

To evaluate the therapeutic effects of [^225^Ac]Ac-PP-F11N, the tumor growth and mean survival time of the immunocompromised A431/CCKBR-tumor bearing nude mice were characterized after the administration of five different doses of purified radiolabeled minigastrin analogue. As displayed in Figure 3a, [^225^Ac]Ac-PP-F11N treatment significantly inhibited the tumor growth in a dose-dependent manner. On day 11, where all mice were still present in all groups, the average tumor volume in the control group reached 0.91 ± 0.36 cm^3^, whereas the average tumor volume in the 30, 45, 60, 90 and 120 kBq [^225^Ac]Ac-PP-F11N-treated mice was reduced to 0.54 ± 0.39 (*p* = 0.0142), 0.31 ± 0.38 (*p* < 0.001), 0.12 ± 0.11 (*p* < 0.001), 0.11 ± 0.05 (*p* < 0.001) and 0.07 ± 0.05 cm^3^ (*p* < 0.001), respectively. In the control group, 11 days after PBS injection, the first mouse had to be euthanized due to exceeding the tumor volume of 1.5 cm^3^ (endpoint), whereas in the 30, 45, 60, 90 and 120 kBq [^225^Ac]Ac-PP-F11N treatment groups the first tumor exceeded 1.5 cm^3^ on day 12, 13, 21, 33 and 44, respectively. During the studies, no body weight loss or other cytotoxicity symptoms were observed in any of the control or treatment groups (Figure 3b and Supplementary Figure S3). Moderately higher increase in the body weight in the control group presumably resulted from fast tumor growth. As shown by Kaplan-Meier curves in Figure 3c, [^225^Ac]Ac-PP-F11N increased the life-span in a dose-dependent manner. The mean survival time in the control group was 17 days, whereas the mean survival in 30, 45, 60, 90 and 120 kBq [^225^Ac]Ac-PP-F11N-treated mice was extended to 22, 27, 34, 44 and 58 days, respectively (Table 1). At termination, there were no significant differences in body or organ weights, general health or anatomy.

### 3.4. Histopathology Analysis and PET/SPECT Imaging

**Figure 4:**
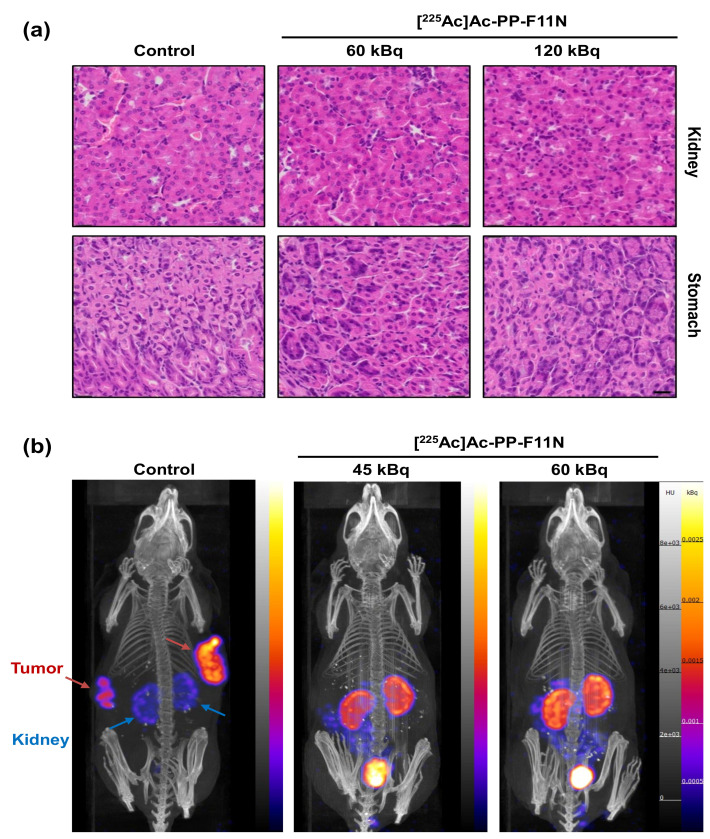
Histological analysis of the kidney and stomach sections an d SPECT/CT imaging after TA (**a**) Representative images of HE stains of the organs isolated from the control, 60, and 120 kBq [^225^Ac]Ac-PP-F11N-treated A431/CCKBR xenografted nude mice. Scale bar: 20 μm. (**b**) SPECT-CT image (left) 20 h post-[^111^In]In-PP-F11N injection of untreated A431/CCKBR tumor-bearing mouse. Red and blue arrows indicate tumors and kidneys, respectively. Middle and right, images taken at 2 h post-[^111^In]In-PP-F11N injection of two mice with no palpable tumors after treatment with 45 and 60 kBq of [^225^Ac]Ac-PP-F11N.

In order to further analyze the toxicity of [^225^Ac]Ac-PP-F11N to healthy organs, kidney and stomach from the control (PBS) and [^225^Ac]Ac-PP-F11N-treated mice were isolated and stained with hematoxylin and eosin (HE) in the late stage of the therapy. The organs derived from the control group were isolated from day 11–26 after PBS injection, whereas from the 60 and 120 kBq [^225^Ac]Ac-PP-F11N treatment groups the organs were dissected between 34 and 49 days post-injection. The HE stains showed no difference among the control and [^225^Ac]Ac-PP-F11N-treated groups (Figure 4a), indicating no signs of acute radiation toxicity during the TAT in the stomach and kidneys. As displayed in Figure 4b, the post-therapy SPECT/CT images of [^111^In]In-PP-F11N in the mice with no visible tumor from 45 and 60 kBq [^225^Ac]Ac-PP-F11N groups indicate no detectable CCKBR positive tumor left. Radioactive signals were only detected in the excretion organs including kidneys and bladder.

## 4. Discussion

Both [^225^Ac]Ac-PP-F11N and [^177^Lu]Lu-PP-F11N showed high internalization efficiency in vitro. Ritler et al. reported [^177^Lu]Lu-PP-F11N internalization rate of 50.2% ± 8.9 after 2 h incubation in CCKBR positive cells recently [16], which is similar to the internalization rate of [^225^Ac]Ac-PP-F11N (45.9% ± 1.6) in our study.

The A431/CCKBR nude mouse tumor model, which was used in the present study, was broadly employed in the previous preclinical studies with radiolabeled minigastrin analogues. In our study, high tumor uptake of [^225^Ac]Ac-PP-F11N and [^177^Lu]Lu-PP-F11N was observed in the A431/CCKBR xenograft nude mice. On the other hand, we observed significant differences in the uptake of radioactivity in liver, kidneys and bones after administration of [^177^Lu]Lu-PP-F11N and [^225^Ac]Ac-PP-F11N. The radiolabeled PP-F11N is a small molecular weight peptide (2.032 kDa), which can be reabsorbed in the proximal tubule after glomerular filtration in the kidney nephron [17], causing renal accumulation of radioactivity. It is reported in several studies that [^225^Ac]Ac’s radioactive daughter nuclides (^221^Fr]Fr, [^217^At]At, [^213^Bi]Bi, [^213^Po]Po to [^209^Tl]Tl, [^209^Pb]Pb—Supplementary Figure S5) are liberated from the metal chelators due to the high recoil energy after decay. Particularly, free bismuth-213 is distributed partially to kidney and bones and contributes to higher activity in kidney. The same holds presumably true for the higher accumulation of radioactivity in the liver [18,19]. However, in the present study, the histological analysis of kidney sections did not show any signs of acute cytotoxicity and suggested that the tested efficacious therapeutic doses of [^225^Ac]Ac-PP-F11N were safe for kidney. In addition, in the recent lumed phase 0a study of [^177^Lu]Lu-PP-F11N, Rottenburger et al. reported relatively low kidney radiation dose in six MTC patients [12]. This suggests that kidneys will not be the dose-limiting organs for TAT. The latter study reported that stomach received the highest radiation dose (median 0.42 Gy/GBq) due to the endogenous CCKBR expression in this organ. Our preclinical histological analysis showed no differences between stomachs isolated from the control and [^225^Ac]Ac-PP-F11N-treated mice indicating that examined by our study therapeutic doses did not induce acute stomach toxicity.

The tissue penetrating range of alpha particles is very short and thus, it is rather unlikely that observed in our study accumulation of radioactivity in mouse bones would cause severe damage to the bone marrow in human. Indeed, the complete response without significant hematological toxicity was previously reported in two patients with prostate cancer after alpha therapy with [^225^Ac]Ac-PSMA-617 [7]. More recently, Sathekge et al. reported therapeutic efficacy of [^225^Ac]Ac-PSMA-617 without significant changes in the leucocyte and platelet count as well as in hemoglobin and serum albumin level in seventeen patients with advanced prostate cancer [20]. Thus, these studies demonstrated the therapeutic potential of TAT without hematological toxicity in the cancer patients. More recently, early treatment with 40 kBq of [^225^Ac]Ac-PSMA-617 prevented liver metastases and led to significant survival benefit in metastatic prostate cancer mouse model [21]. Similarly, in our preclinical study, injection of different doses (30–120 kBq) of [^225^Ac]Ac-PP-F11N showed dose-dependent inhibition of tumor growth as well as extended mean survival time and suggest further development of safe and efficacious α particle therapy. In addition, the efficacious therapeutic doses of [^225^Ac]Ac-PP-F11N analyzed in the present study did not cause any toxic symptoms including body weight loss or acute radiation syndrome. Nevertheless, long-term toxicity study of [^225^Ac]Ac-PP-F11N warrants further investigation.

## 5. Conclusions

Our study demonstrates efficacious and safe targeted alpha particle therapy with actinium-225 labeled minigastrin analogue in the A431/CCKBR tumor-xenograft nude mouse model and recommends further development of [^225^Ac]Ac-PP-F11N for the treatment of CCKBR-positive cancers.

## 6. Patents

Part of the results of this study has been used for the patent application.

## Figures and Tables

**Table 1:** Mean survival times of [^225^Ac]Ac-PP-F11N-treated A431/CCKBR tumor-bearing nude mice.

Treatment [^225^Ac]Ac-PP-F11N	Mean Survival (days)	*p*-Value
Control	17	−
30 kBq	22	0.36
45 kBq	27	0.04
60 kBq	34	<0.0001
90 kBq	44	<0.0001
120 kBq	58	<0.0001

## Supplementary Materials

The following are available online at https://www.mdpi.com/1999-4923/12/11/1088/s1, Figure S1: Analysis of radiochemical purity of radiolabeled PP-F11N. (a) Thin-layer chromatography of free [^225^Ac]Ac with and without EDTA, and [^225^Ac]Ac-PP-F11N in radioactive fractions 24 hours after separation. The samples were analyzed by Cyclone® Plus Storage Phosphor System (PerkinElmer, 2200 Warrenville Road Downers Grove, IL, USA, 60515) and the images were quantified by OptiQuant™ Image Analysis Software (PerkinElmer, 2200 Warrenville Road Downers Grove, IL 60515). The [^225^Ac]Ac-PP-F11N yield in the fraction 12, 13 and 14 was 88, 97, and 96 %, respectively. (b) A reverse-phase HPLC chromatograms for Lu-177 and In-111 labeled PP-F11N. Low panel, a UV profile (A 220 nm) of unlabeled PP-F11N. Retention times; 5.50, 5.53 and 4.70 min for ^177^Lu—, ^111^In-labeled and unlabeled PP-F11N. CPS; Counts per second. mAU; milli-absorbance unit, Figure S2: Cellular uptake of [^177^Lu]Lu-PP-F11N in A431/CCKBR cells. Internalized and membrane-bound activity after 2 h treatment with [^177^Lu]Lu-PP-F11N in A431/CCKBR cells. Bars represent mean ± SD, Figure S3: Tumor growth curves of [^225^Ac]Ac-PP-F11N-treated mice. After tumor implantation, PBS or 30, 45, 60, 90 and 120 kBq of purified [^225^Ac]Ac-PP-F11N was administrated into the A431/CCKBR tumor-bearing nude mice as indicated. Graphs show the tumor volume of each mouse and means ± SD in different treatment groups, Figure S4: Body weight changes in [^225^Ac]Ac-PP-F11N-treated mice. After tumor implantation, PBS or 30, 45, 60, 90 and 120 kBq of purified [^225^Ac]Ac-PP-F11N was administrated into A431/CCKBR tumor-bearing nude mouse groups, as indicated. Graphs show the body weight of each mouse and means ± SD in different treatment group, Figure S5: Decay chain of actinium-225 [7], Table S1: Biodistribution of [^225^Ac]Ac-PP-F11N in different organs dissected from A431/CCKBR xenografted nude mice, Table S2: Tumor to kidney and tumor to stomach radioactivity ratios in [^225^Ac]Ac-PP-F11N-treated A431/CCKBR xenografted nude mice.
