# Supplementary material for: Evaluation of Actinium-225 Labeled Minigastrin Analogue [225Ac]Ac-DOTA-PP-F11N for Targeted Alpha Particle Therapy
Source: Pharmaceutics. 2020 Nov 12;12(11):1088. doi: 10.3390/pharmaceutics12111088 (PMC7696055; doi:10.3390/pharmaceutics12111088)
Supplement: Supplementary file 1 [file pharmaceutics-12-01088-s001.pdf]

Supplementary Figure S1.

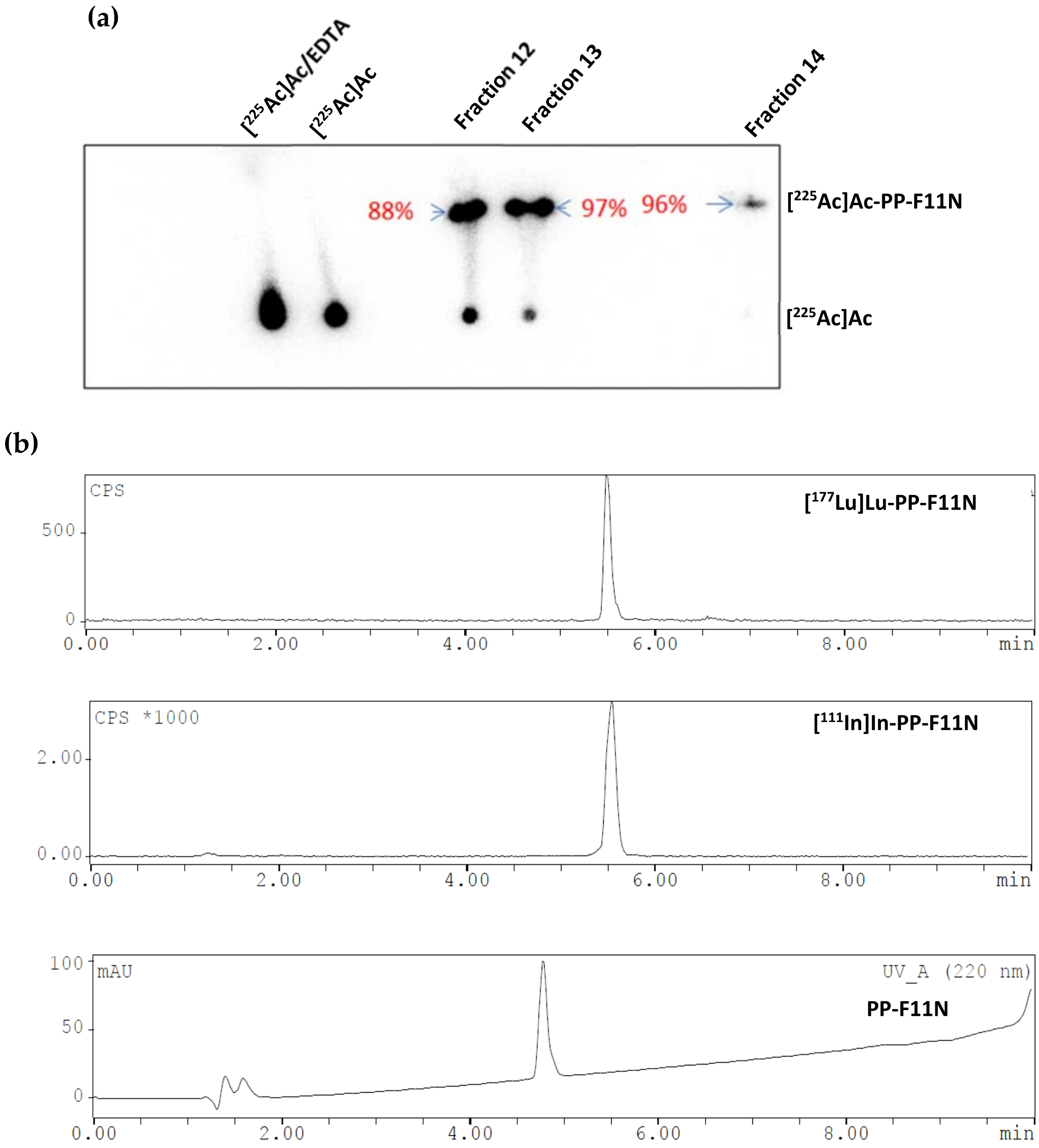

Supplementary Figure S1. Analysis of radiochemical purity of radiolabeled PP-F11N (**a**) Thin-layer chromatography of free [^{225}Ac]Ac with and without EDTA, and [^{225}Ac]Ac-PP-F11N in radioactive fractions 24 hours after separation. The samples were analyzed by Cyclone® Plus Storage Phosphor System (PerkinElmer, 2200 Warrenville Road Downers Grove, IL 60515) and the images were quantified by OptiQuant™ Image Analysis Software (PerkinElmer, 2200 Warrenville Road Downers Grove, IL 60515). The [^{225}Ac]Ac-PP-F11N yield in the fraction 12, 13 and 14 was 88, 97 and 96 %, respectively. (**b**) A reverse-phase HPLC chromatograms for Lu-177 and In-111 labeled PP-F11N. Low panel, a UV profile (A 220 nm) of unlabeled PP-F11N. Retention times; 5.50, 5.53 and 4.70 min for ^{177}Lu-, ^{111}In-labeled and unlabeled PP-F11N. CPS; Counts per second. mAU; milli-absorbance unit

Supplementary Figure S2.

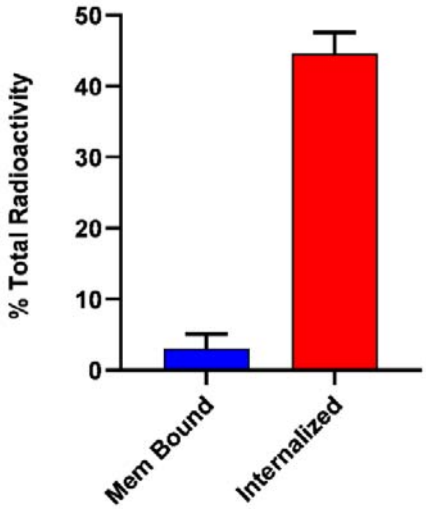

Supplementary Figure S2. Cellular uptake of [^{177}Lu]Lu-PP-F11N in A431/CCKBR cells. Internalized and membrane-bound activity after 2 h treatment with [^{177}Lu]Lu-PP-F11N in A431/CCKBR cells. Bars represent mean ± SD.

Supplementary Figure S3.

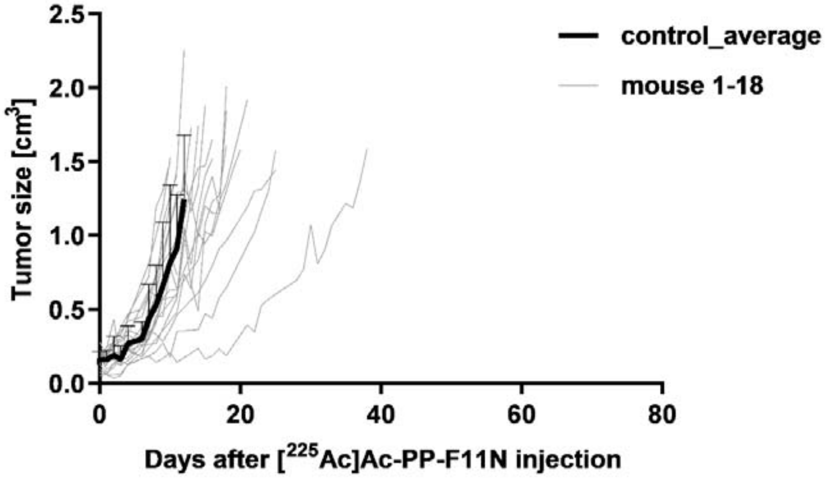

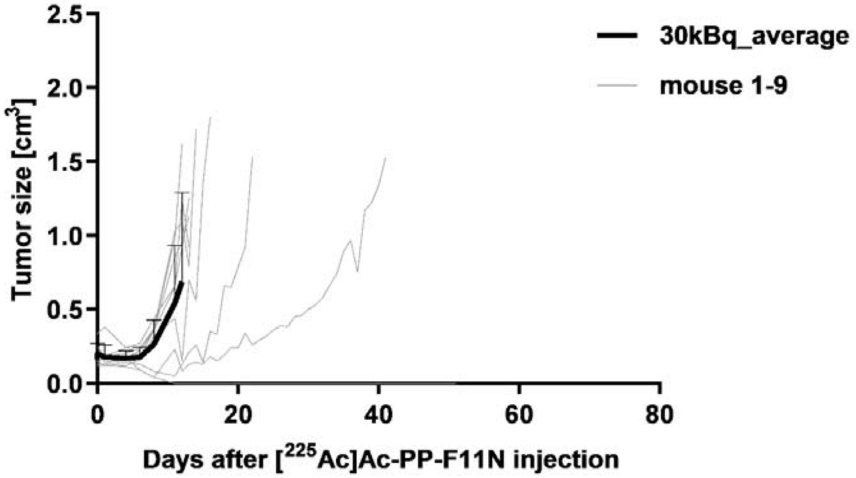

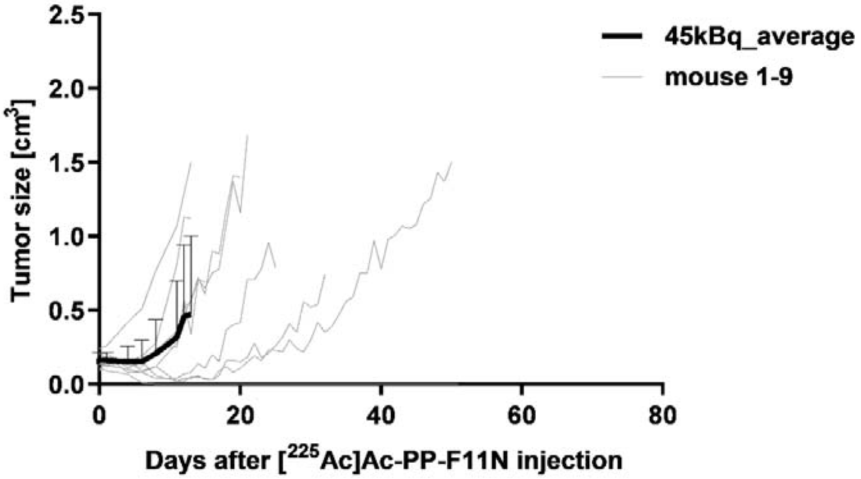

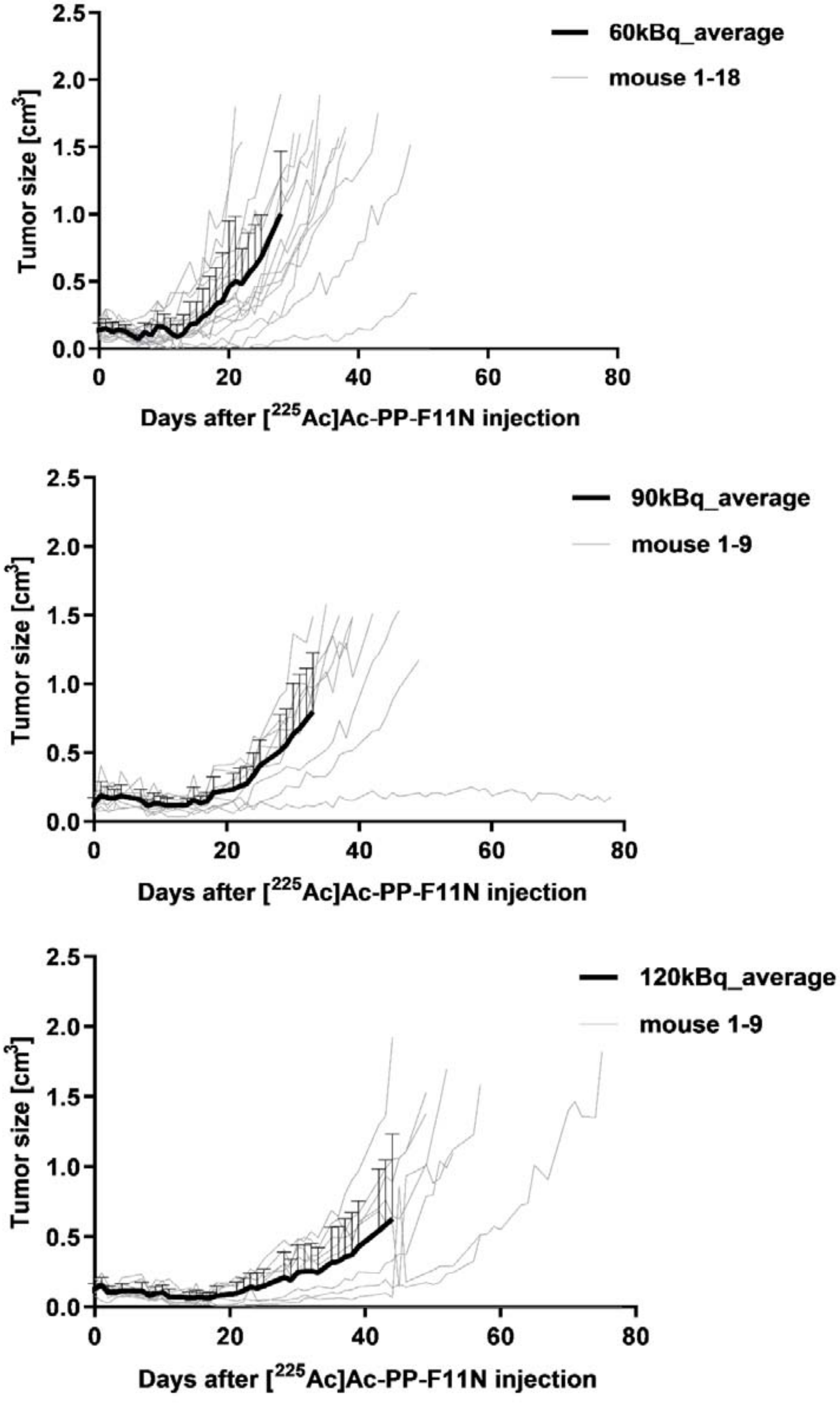

Supplementary Figure S3. Tumor growth curves of the [^{225}Ac]Ac-PP-F11N-treated mice. After tumor implantation, PBS or 30, 45, 60, 90 and 120 kBq of purified [^{225}Ac]Ac-PP-F11N was administrated into the A431/CCKBR tumor-bearing nude mice as indicated. Graphs show the tumor volume of each mouse and means ± SD in different treatment groups.

Supplementary Figure S4.

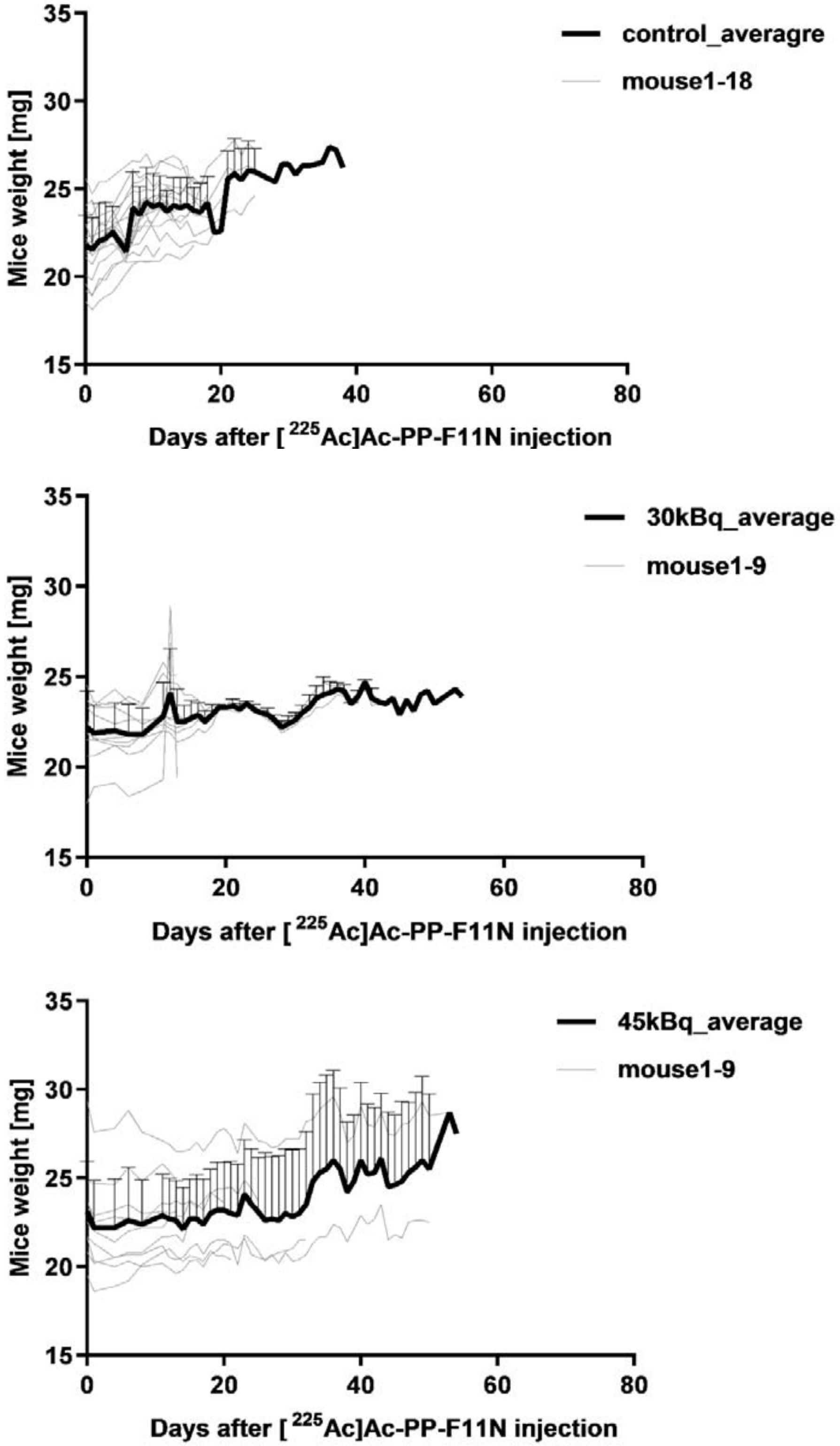

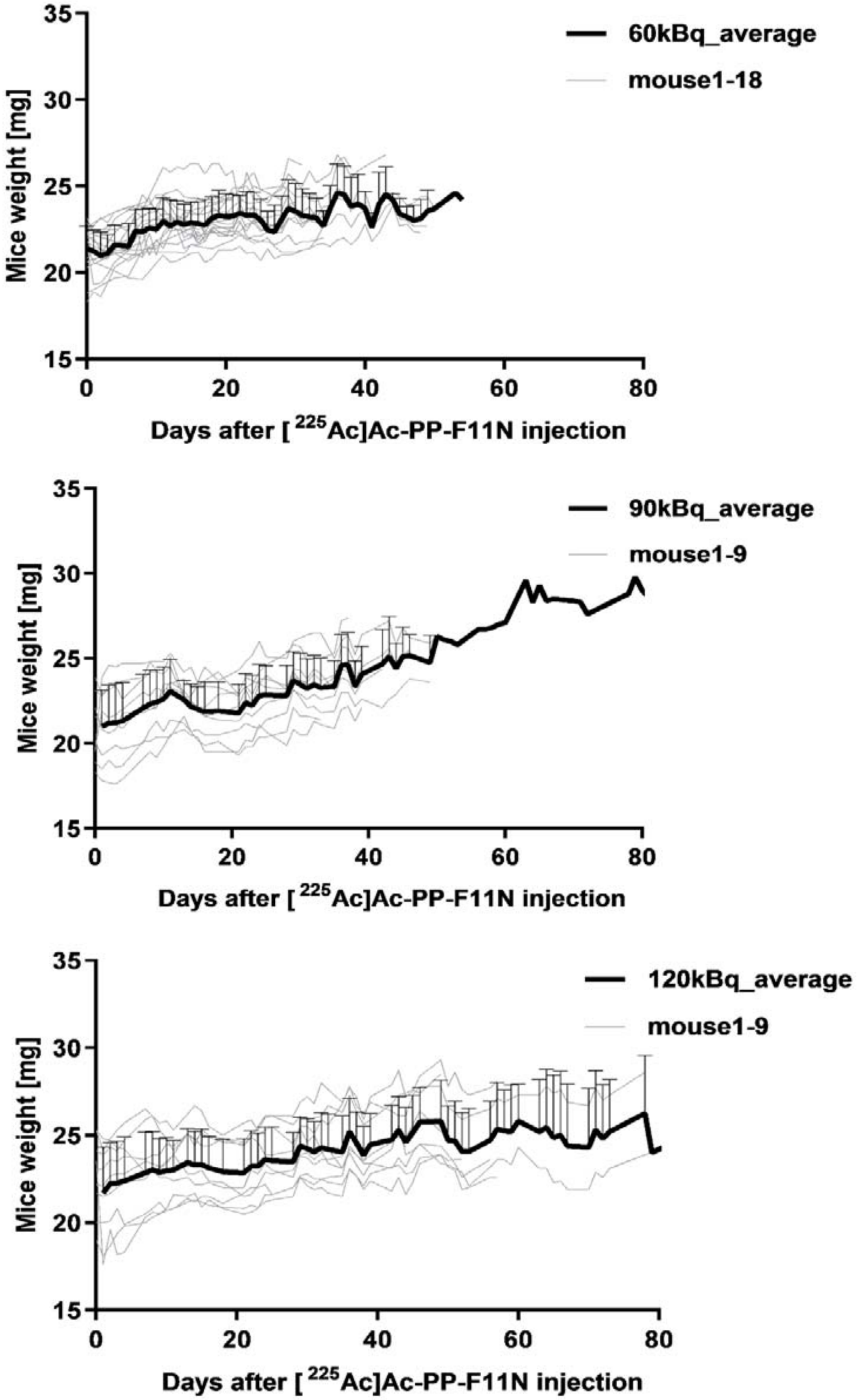

Supplementary Figure S4. Body weight changes in the [^{225}Ac]Ac-PP-F11N-treated mice. After tumor implantation, PBS or 30, 45, 60, 90 and 120 kBq of purified [^{225}Ac]Ac-PP-F11N was administrated into A431/CCKBR tumor-bearing nude mouse groups, as indicated. Graphs show the body weight of each mouse and means ± SD in different treatment groups.

Supplementary Figure S5.

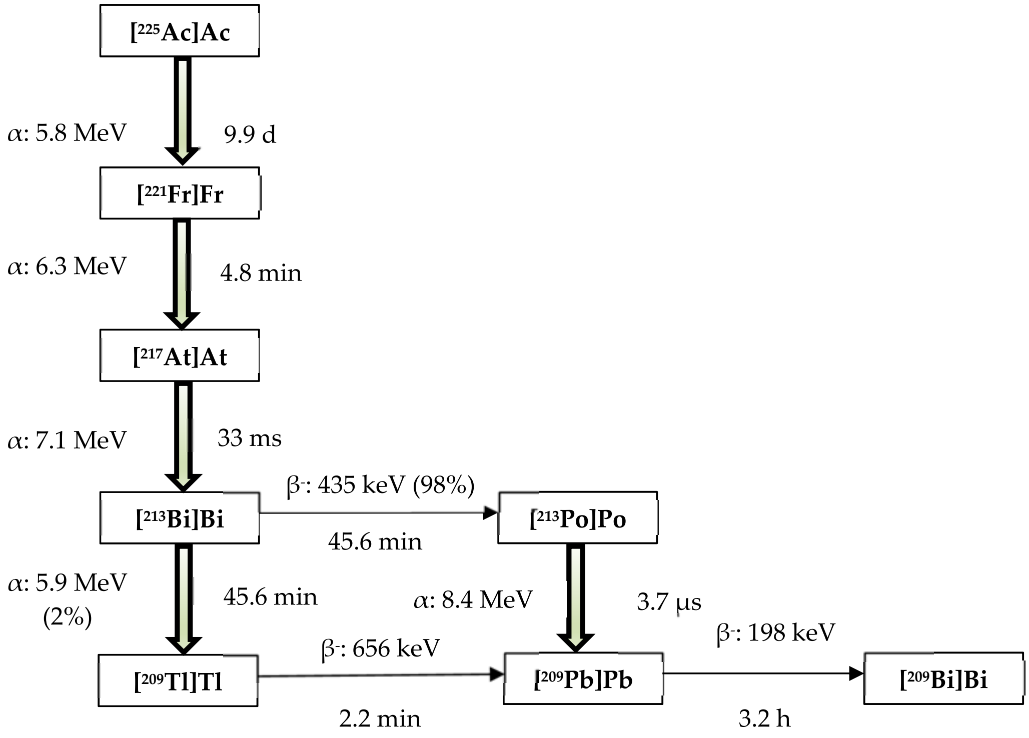

Supplementary Figure S5. Decay chain of actinium-225 [7].

Supplementary Table S1.

Time after [225Ac]Ac-PP-F11N injection	% Total injected radioactivity per gram of tissue [% i.A./g]												
	Tumor	Kidney	Stomach	Liver	Bone	Lung	Spleen	Intestines	Pancreas	Blood	Heart	Muscle	Brain
1 h	12.95 ± 4.962	8.14 ± 1.741	1.46 ± 0.58	1.04 ± 0.219	1.70 ± 0.219	0.35 ± 0.061	0.29 ± 0.045	0.32 ± 0.098	0.28 ± 0.031	0.28 ± 0.057	0.38 ± 0.042	0.21 ± 0.150	0.02 ± 0.003
4 h	11.19 ± 1.894	4.20 ± 0.641	1.31 ± 0.297	1.44 ± 0.115	1.43 ± 0.295	0.09 ± 0.008	0.16 ± 0.010	0.14 ± 0.030	0.16 ± 0.022	0.03 ± 0.004	0.20 ± 0.044	0.04 ± 0.015	0.01 ± 0.003
24 h	7.24 ± 1.807	3.80 ± 0.457	1.18 ± 0.687	1.80 ± 0.207	1.08 ± 0.058	0.11 ± 0.006	0.21 ± 0.030	0.11 ± 0.016	0.19 ± 0.033	0.02 ±0.005	0.11 ± 0.019	0.03 ± 0.006	0.01 ± 0.002
48 h	5.77 ± 1.818	2.73 ± 0.585	0.82 ± 0.226	1.71 ± 0.216	0.72 ± 0.087	0.10 ± 0.013	0.19 ± 0.043	0.10 ± 0.010	0.18 ± 0.043	0.02 ± 0.004	0.10 ± 0.021	0.03 ± 0.005	0.01 ± 0.001
1 week	4.47 ± 2.468	1.23 ± 0.194	0.39 ± 0.092	1.84 ± 0.210	0.91 ± 0.298	0.07 ± 0.024	0.15 ± 0.015	0.04 ± 0.015	0.11 ± 0.018	0.01 ± 0.005	0.06 ± 0.024	0.02 ± 0.005	0.01 ± 0.007

Supplementary Table S1. Biodistribution of [^{225}Ac]Ac-PP-F11N in different organs dissected from A431/CCKBR xenografted nude mice.

Supplementary Table S2.

Time after [^{225}Ac]Ac-PP-F11N injection	Ratio: Tumor/ Kidney	Ratio: Tumor/ Stomach
1 h	1.59	8.85
4 h	2.66	8.52
24 h	1.90	6.13
48 h	2.11	7.03
1 week	3.64	11.49

Supplementary Table S2. Tumor to kidney and tumor to stomach radioactivity ratios in [^{225}Ac]Ac-PP-F11N-treated A431/CCKBR xenografted nude mice.
